# A conserved role of the RSC chromatin remodeler in the establishment of nucleosome-depleted regions

**DOI:** 10.1007/s00294-016-0642-y

**Published:** 2016-08-24

**Authors:** Carlo Yague-Sanz, Enrique Vázquez, Mar Sánchez, Francisco Antequera, Damien Hermand

**Affiliations:** 1 URPHYM-GEMO, Namur Research College (NARC), The University of Namur, 5000 Namur, Belgium; 2 Instituto de Biología Funcional y Genómica, Consejo Superior de Investigaciones Científicas (CSIC)/Universidad de Salamanca, Campus Miguel de Unamuno, 37007 Salamanca, Spain

**Keywords:** Yeast, Chromatin, RSC, Nucleosome, Mitosis

## Abstract

The occupancy of nucleosomes governs access to the eukaryotic genomes and results from a combination of biophysical features and the effect of ATP-dependent remodelling complexes. Most promoter regions show a conserved pattern characterized by a nucleosome-depleted region (NDR) flanked by nucleosomal arrays. The conserved RSC remodeler was reported to be critical to establish NDR in vivo in budding yeast but other evidences suggested that this activity may not be conserved in fission yeast. By reanalysing and expanding previously published data, we propose that NDR formation requires, at least partially, RSC in both yeast species. We also discuss the most prominent biological role of RSC and the possibility that non-essential subunits do not define alternate versions of the complex.

## Introduction

A fundamentally different logic of gene regulation between prokaryotes and eukaryotes was previously proposed based on the existence of chromatin in the latter, which results in a closed, less accessible genome (Struhl 1999). Despite the fact that this view may be too simplistic—there are structural proteins associated with the DNA in prokaryotes (Anuchin et al. 2011) and eukaryotes use more repressors than anticipated (Kemmeren et al. 2014)—the general concept still stands true (Estrada et al. 2016). Strikingly, the human genome harbours the blueprint of about 200 highly specialized cell types characterized by very different morphology, metabolism and capacities including, for example, neurons, hepatocytes or gametes. By comparison, most prokaryotes have a limited range of cellular states. Therefore, the invention of chromatin may have been pivotal for the emergence of highly differentiated cell types, most likely because the expression of specific programmes must be tightly regulated to allow diverse and sometimes antagonistic differentiated states to co-exist. For example, yeast differentiation during gametogenesis must be very strictly limited to diploid cells to avoid massive cell death resulting from haploid meiosis and recent data support that chromatin-based mechanisms play a key role in that process (van Werven et al. 2012). The understanding of how chromatin is established and how it contributes with most, if not all nuclear processes including transcription, DNA replication, DNA repair, recombination or chromosome segregation therefore constitutes an outstanding focus in current biology. In that context, an important and long-standing question is to decipher how nucleosomes, which constitute the basic unit of chromatin, are positioned genome-wide. There have been abundant debates about what dictates the position of nucleosomes with models fully relying on biophysics—the position is DNA encoded—and a model encompassing a layer of active, ATP-dependent modelling of the chromatin template. The reader is redirected to excellent reviews addressing these issues (Korber 2012; Lieleg et al. 2015; Struhl and Segal 2013). In the meantime, the discovery of a large set of chromatin remodelers supported the second possibility and the development of more refined nucleosome occupancy maps in various species led to the discovery of general features of nucleosome positioning along the eukaryotic genomes. Particularly, nearby the promoter, there is often a stereotypical organization just upstream of the transcription start site (TSS) with a large nucleosome-depleted region (NDR) flanked by highly positioned nucleosomes referred to as the +1 and the −1. From these two landmarks, arrays of regularly spaced nucleosomes often extend. The remodel the structure of chromatin (RSC) complex has a specific role in the generation of NDR in budding yeast (Badis et al. 2008; Hartley and Madhani 2009; Parnell et al. 2008; Wippo et al. 2011). RSC is an abundant and essential paralog of the canonical SWI/SNF remodeler (Cairns et al. 1996) that contains a DNA-dependent ATPase (Sth1 in budding yeast, Snf21 in fission yeast) that translocates DNA and allows to shift nucleosome positions, or even completely eject nucleosomes. Importantly, RSC show compositional and functional differences between budding and fission yeasts (Monahan et al. 2008) and it was suggested that RSC is not required for NDR formation in the latter (Pointner et al. 2012). In addition, not all subunit of RSC are essential, which may indicate that subcomplexes exist and may have specialized functions. Finally, it is unclear what is the most prominent biological role of RSC, in other words which nuclear process is mainly affected upon RSC inactivation and results in lethality. Here we briefly comment on these issues by reanalysing previously published data from budding yeast and by building on our recent work in fission yeast.

### Defects in the RSC remodeler affects NDR formation in both budding yeast and fission yeast

**Fig. 1:**
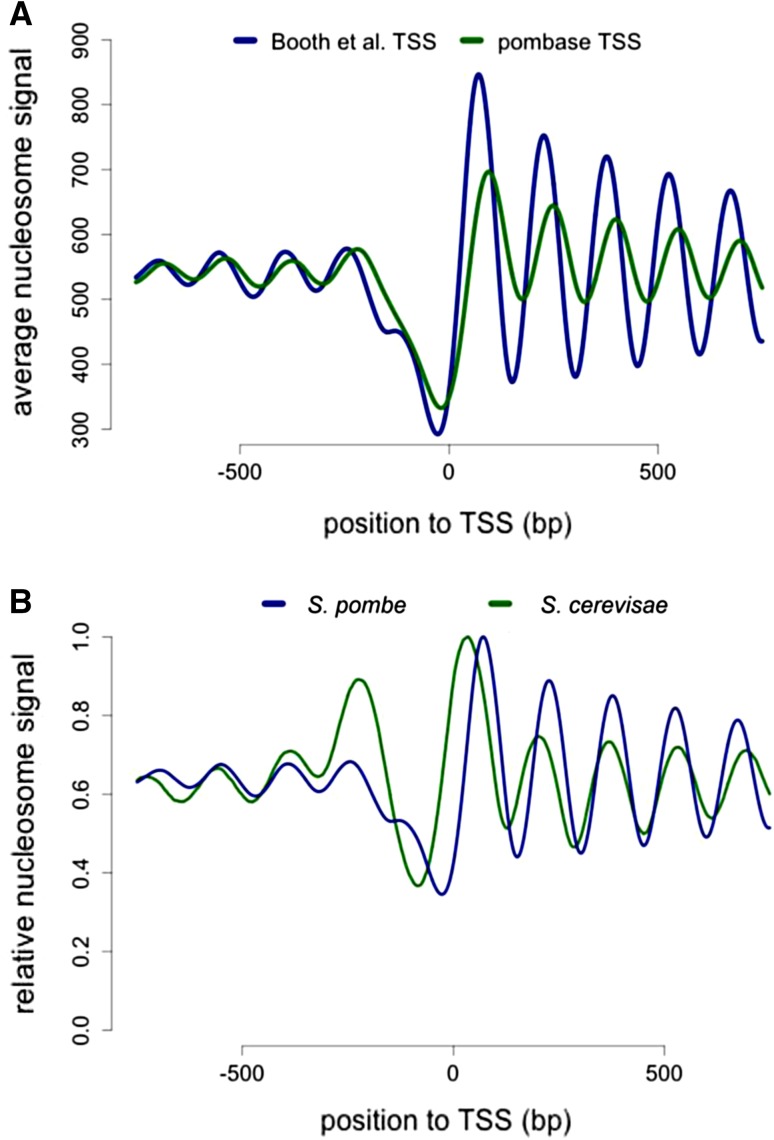
Average nucleosome occupancy nearby the TSS in fission and budding yeasts. **a** Average nucleosome signals centered on two different fission yeast TSS annotations. **b** Average nucleosome signals centered on TSS in fission yeast and budding yeasts

One of the early difficulties in comparing the two model yeast species (the budding yeast *Saccharomyces cerevisiae*, hereafter *S. cerevisiae,* and the fission yeast *Schizosaccharomyces pombe*, hereafter *S. pombe*) was the very poor annotation of the transcription start sites in *S. pombe*. This is an issue when plotting the average nucleosome signal obtained from MNase-Seq experiments to transcripts coordinates. Very recently, several groups (Booth et al. 2016; Eser et al. 2016; Li et al. 2015) have used different approaches to address that issue and Fig. 1a shows a comparison of the average nucleosome signal when plotted to the Pombase annotation (the reference of the community working on fission yeast) and the most recent work performed by the Lis laboratory using precision run-on 5′ cap sequencing (PRO-cap) (Booth et al. 2016), which corresponds to the annotation used in this manuscript. Comparing the overlay of MNase-Seq data after alignment at the TSS between the two yeast species confirms previous observations from the pioneer work of the Korber laboratory in fission yeast, namely the absence of a clearly positioned −1 nucleosome and shorter nucleosome spacing in *S. pombe* (Lantermann et al. 2009). In addition, the +1 nucleosome is positioned further away from the TSS and there are clear, albeit weak nucleosome arrays upstream of NDR in fission yeast (Fig. 1b). Importantly, the low amplitude of the peaks and the absence of a positioned −1 nucleosome were previously shown to result from the larger variation in the size of individual NDRs in fission yeast (Soriano et al. 2013). Therefore, nucleosomal arrays emanate bidirectionally from the NDRs in fission yeast as well.

It is now well established that the ablation of RSC activity in budding yeast strongly affects the majority of NDRs (Badis et al. 2008; Hartley and Madhani 2009; Parnell et al. 2008). Recent data further confirmed that in the inactivation of RSC (resulting from switching-off the expression of *rsc8*) leads to upstream and downstream nucleosomal arrays to shift to and eventually occlude the NDR. This work also supports the idea that phasing patterns reflect the resultant of phasing signals emanating from neighbouring NDRs (Ganguli et al. 2014). Most recently, it was reported by the Cairns laboratory that RSC and ISW1 have functional antagonism, which is supported by the fact that the gain in nucleosome occupancy in *rsc* mutant is attenuated by the additional inactivation of ISW1 (Parnell et al. 2015).

In the fission yeast, the RSC complex was also associated with the generation of NDR in the context of heterochromatin. Indeed the deletion of *rsc1* that encodes a non-essential subunit of RSC suppresses the requirement of the histone deacetylase (HDAC) Clr3 for NDR elimination (Garcia et al. 2010), which indirectly supports that RSC is responsible for acetylation-dependent NDR formation in that species. However, that study did not expand to euchromatin. Finally, it was reported that CHD1 remodelers, Hrp1 and Hrp3 are required in fission yeast to link nucleosomal arrays to most TSS (Pointner et al. 2012). In the same study, the role of RSC in nucleosome positioning was also analysed using the only conditional (thermosensitive) mutant allele available for the gene *snf21* that encodes the catalytic subunit of RSC (Yamada et al. 2008). Unexpectedly, no effect on nucleosome positioning around TSS was obvious upon thermal inactivation of the mutant, which suggested that RSC plays no role in NDR formation in *S. pombe*.

Our recent work identified RSC as a key downstream effector of a cascade controlling the level of acetylation around the NDR of *ste11*, which encodes the master regulator of gametogenesis in *S. pombe* (Anandhakumar et al. 2013; Cassart et al. 2012; Coudreuse et al. 2010; Devos et al. 2015). Deletion of non-essential subunits of RSC or transcriptional switch-off of *snf21* both impede *ste11* expression and correlate with higher nucleosome occupancy at the *ste11* NDR (Materne et al. 2015, 2016). This effect led us to analyse the genome-wide effect of both RSC mutants on nucleosome positioning using MNase-Seq, which allows us to assess the conservation of the chromatin remodelling function of the RSC complex in budding and fission yeast.

**Fig. 2:**
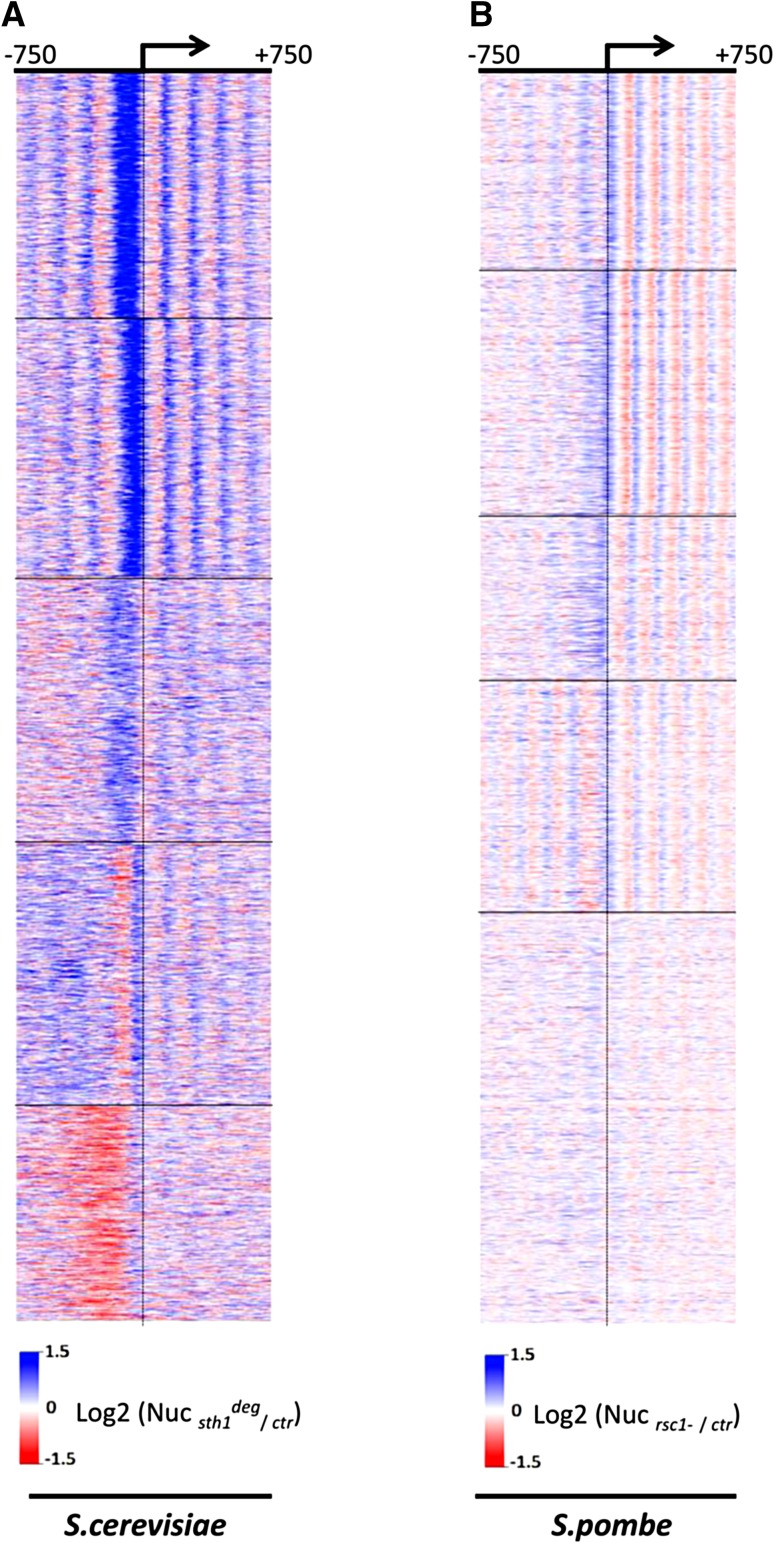
Defects in the RSC remodeler affects NDR formation in both budding yeast and fission yeast. **a** The profile of nucleosome occupancy ratios between budding yeast *sth1* degron (*sth1* ^*deg*^) and control strains is presented as an heatmap, where *blue* represents a gain in nucleosome occupancy and *red* represents a loss within a region ranging from −750 bp to +750 bp around the TSS at single nucleotide resolution. *Rows* represent genes and are organized into five groups by* k*-means clustering. **b** Same as in **a**, except that the fission yeast *rsc1* deletion mutant (*rsc1Δ*) and control strains are presented

We have reanalysed the data presented in the Parnell et al. (2015) paper with the following modifications compared to the published work. First, no filtering for specific gene organization was applied. Second, the data are presented at single base pair resolution rather that within 50 bp windows relative to the TSS as done before. This was made possible as we used the MNase-Seq data rather than the Agilent 244 K mircoarrays used for the main figures of the original study. Third, the TSS annotation was obtained from a different source (Ganguli et al. 2014). Figure 2a shows the profile of nucleosome occupancy ratios between the *sth1* degron (a strain that allows rapid and conditional degradation of the Sth1 protein RSC subunit) and control strains ±750 bp relative to the TSS. Genes were organized into clusters based on their *rsc*–*/*RSC+ ratio. As reported in the published work, a leftward shift in nucleosome positions over the transcribed region is observed, confirming that nucleosomal arrays emanate from the NDR flanking nucleosomes. Most likely thus, NDR filling reflects encroachment by the flanking −1 and +1 nucleosomes, rather than insertion of an additional nucleosome within the NDR region as previously discussed (Ganguli et al. 2014).

**Fig. 3:**
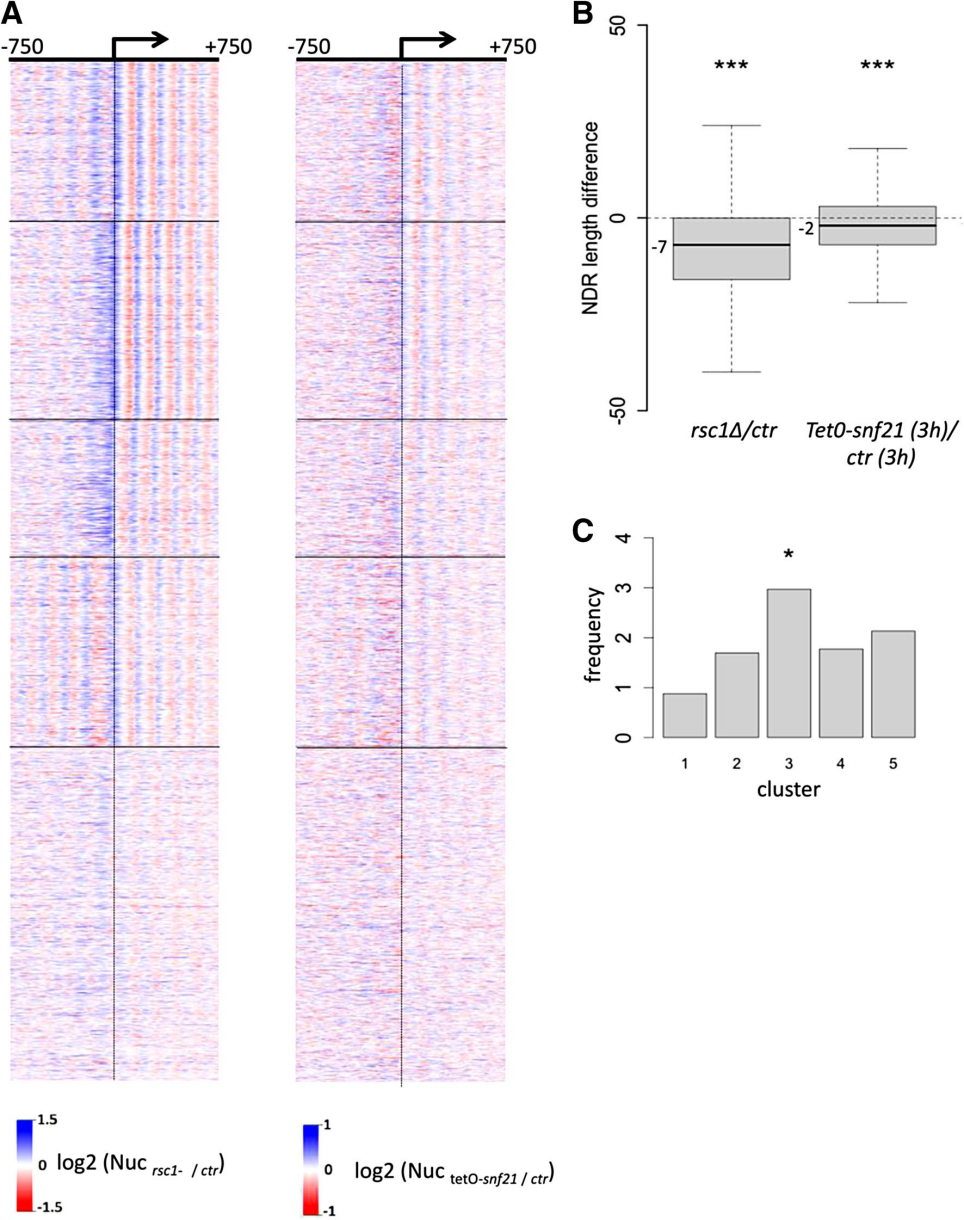
Comparison of the fission yeast *rsc1* deletion mutant and the *snf21* switch-off strains. **a** The profile of nucleosome occupancy ratios between fission yeast *rsc1* deletion mutant (*rsc1Δ*) and control strains on the *left panel* (note that this *panel* is identical to Fig. 2b and repeated here for clarity), and between the fission yeast *snf21* switch-off mutant [*tetO*-*snf21,* 3 h of inhibition, (Materne et al. 2015)] and control strains on the *right panel* are presented as a heatmap, where *blue* represents a gain in nucleosome occupancy and *red* represents a loss within a region ranging from −750 bp to +750 bp around the TSS at single nucleotide resolution. *Rows* represent genes and are organized into five groups by* k*-means clustering. **b** *Box plot* representing the difference in NDR length between the *rsc1Δ* and *tetO*-*snf21* strains and the corresponding wt strains. Statistical significance was calculated by a one-sample Wilcoxon test (*pval* <0.01). **c** Frequency of genes downregulated (log2 fold change < −0.5, based on (Monahan et al. 2008) in *rsc1Δ* strain sorted by *cluster*. *Cluster 3* includes the *ste11* gene and is enriched (Fisher’s exact test *P* value <0.05) for genes whose expression is downregulated in the *rsc1Δ* strain

We next applied identical analyses to the data obtained in a fission yeast *rsc1* mutant. It should be noted straight away that Rsc1 is a non-essential subunit of RSC while the work done in budding yeast targeted the gene encoding the catalytic subunit. Nonetheless, the general picture obtained when *rsc1* is absent (Fig. 2b) is reminiscent of the budding yeast data (Fig. 2a). Compared to budding yeast, the shortening of NDR is observed with a slight shift towards the TSS that recalls the shift of the position of the +1 nucleosome between budding and fission yeast (Fig. 1b). In addition, the leftward shift in nucleosome positions over the transcribed region is also obvious in most clusters. These data suggest that similarly to budding yeast, RSC also play an important conserved role in the establishment of NDRs in fission yeast in contrast to previous conclusions (Pointner et al. 2012). However, it is important to keep in mind that the previous study relies on a *ts* allele of *snf21*. As rightly pointed by the authors in their manuscript, it is possible that the inactivation of the *snf21*-*ts* was not complete despite the fact that the strain has obvious phenotypes (see below), somehow masking an effect of most NDR. Supporting this possibility, we report here that while switching-off *snf21* expression using a Tet-off system eventually results in cell death on plates (Materne et al. 2015), it has a weak genome-wide effect on NDR formation when a short time point is used (Fig. 3a). This suggests that lowering RSC activity by switching-off the transcription of the *snf21* gene (with about 35 % of the *snf21* mRNA left) may only affect the most sensitive RSC-dependent processes. These data claim for the generation of a much more efficient switch-off system that could quickly deplete the vast majority of the Snf21 protein pool in the cell.

## A single RSC complex containing non-essential subunits likely fulfils all RSC functions

Interestingly, the analysis of *rsc1* deletion in fission yeast reveals a genome-wide effect on NDR, yet the effect appears milder than the inactivation of the catalytic subunit in *S. cerevisiae* (compare Fig. 2a, b). However, the shrinkage of NDR when *rsc1* is deleted is statistically significant (one-sample Wilcoxon test *p* < 0.01), Fig. 3b). To us, these data do not support that the non-essential subunits constitute an RSC submodule with specific functions but rather indicates that within RSC, the non-essential subunits may have a less prominent structural role than essential subunits. In line with this, all the phenotypes reported when inactivating RSC, including chromosomal segregation defects, and sensitivity to drugs, are shared to various degrees by all mutants (Monahan et al. 2008).

## A key role of RSC in mitotic chromosome condensation

Despite the genome-wide defect observed in NDR formation (Fig. 2b), the *rsc1* mutant has a subtle impact on steady-state transcription. Expression alteration effects in either direction were seen for only 1.4 % of *S pombe* genes in the *rsc1* mutant and other non-essential subunit similarly affect the transcriptome (Monahan et al. 2008). Interestingly, the third cluster in Fig. 2b harbours the highest frequency of genes affected in the *rsc1* mutant (Fig. 3c), including *ste11* (Materne et al. 2015, 2016). That cluster is characterized by an increase in occupancy over a broad region upstream of the TSS that may be typical of highly regulated genes relying on larger regulatory sequences, as typically seen for *ste11* (Anandhakumar et al. 2013). Nonetheless, the main phenotypes of the *rsc1* mutant, and the *snf21*-*ts* mutant for that matter, are cell elongation associated with chromosomes segregation defects. Although these phenotypes may result from the reduced expression of specific genes, a recent study rather points to a direct role of RSC and nucleosome eviction in condensin loading and chromosome condensation (Toselli-Mollereau et al. 2016). A genetic screen for functional partners of condensin in fission yeast (synthetic lethality with *cut3*-*477* that encodes a condensin ATPase subunit) identified alleles of *arp9* and *snf21* that both showed high frequency of chromatin bridges in anaphase (Robellet et al. 2014). Further analyses revealed the preferred localization of condensin at, or near NDR and that increased nucleosome occupancy upon RSC downregulation is sufficient to decrease condensin binding. These data point to a prominent role of RSC is establishing the landscape of condensin binding during mitosis by establishing NDR.

In conclusion it appears that the role of RSC in NDR formation is conserved between both *S. pombe* and *S. cerevisiae*. The milder effect observed with the *rsc1* mutant in fission yeast may be caused by (1) the fact that this subunit is not essential and its deletion could only partially impair RSC activity; (2) the presence of an additional remodeler involved in NDR formation; (3) the fact that *S. pombe* lacks ISW1-type remodelers that are known to oppose the action of RSC in NDR formation in budding yeast (Parnell et al. 2008, 2015). Future work will clarify this issue. Biologically, the most prominent role of RSC may be to maintain proper chromosome segregation and may extend to kinetochore function, sister chromatid cohesion and DNA repair, in addition to its role in promoting transcription (Cao et al. 1997; Hsu et al. 2003; Huang et al. 2004; Shim et al. 2007).

## Bioinformatic analyses

TSS annotations for *S. pombe* were obtained from pombase (http://www.pombase.org/, ASM294v2.26) and (Booth et al. 2016). TSS annotation for *S. cerevisiae* was obtained from (Ganguli et al. 2014). Nucleosome genome-wide occupancy profiles at 1 bp resolution were generated as described (Bauer and Hermand 2012; Drogat et al. 2012; Lenglez et al. 2010; Materne et al. 2015) using DANPOS for the *S. pombe* data or directly downloaded from GEO (supplementary of GSE65593) for the *S. cerevisiae* data (Parnell et al. 2015).

Nucleosome occupancy ratios were computed as the log2 ratio between treatment and control profiles centered at TSS for all protein coding genes. A pseudocount of +1 was added to both the numerator and the denominator to avoid division by zero. Clustering of the nucleosome occupancy ratio was made using the *kmeans()* function in base R with default parameters and *k* = 5 and visualized with the *heatmap.2()* function from the “gplots” package.

Nucleosome positions were computed for each dataset as the local maximum of nucleosome occupancy in a 100 bp window using the *localMaximum()* function from the “MassSpecWavelet” package. Promoter NDR length is computed as the distance in bases between the position of the first nucleosome before the TSS and the first nucleosome after the TSS. This metric was computed for mutants and wild type strains and subtracted accordingly to obtain the NDR length difference as in Fig. 3b.

## Data access

The nucleosome sequencing data are available in the GEO database under the accession numbers GSE84912 (*S. pombe* datasets) and GSE65593 (*S. cerevisiae* datasets).

## References

[CR1] Anandhakumar J, Fauquenoy S, Materne P, Migeot V, Hermand D (2013). Regulation of entry into gametogenesis by Ste11: the endless game. Biochem Soc Trans.

[CR2] Anuchin AM, Goncharenko AV, Demidenok OI, Kaprel’iants AS (2011). Histone-like proteins of bacteria (review). Prikl Biokhim Mikrobiol.

[CR3] Badis G, Chan ET, van Bakel H, Pena-Castillo L, Tillo D, Tsui K, Carlson CD, Gossett AJ, Hasinoff MJ, Warren CL (2008). A library of yeast transcription factor motifs reveals a widespread function for Rsc3 in targeting nucleosome exclusion at promoters. Mol Cell.

[CR4] Bauer F, Hermand D (2012). A coordinated codon-dependent regulation of translation by Elongator. Cell Cycle.

[CR5] Booth GT, Wang IX, Cheung VG, Lis JT (2016). Divergence of a conserved elongation factor and transcription regulation in budding and fission yeast. Genome Res.

[CR6] Cairns BR, Lorch Y, Li Y, Zhang M, Lacomis L, Erdjument-Bromage H, Tempst P, Du J, Laurent B, Kornberg RD (1996). RSC, an essential, abundant chromatin-remodeling complex. Cell.

[CR7] Cao Y, Cairns BR, Kornberg RD, Laurent BC (1997). Sfh1p, a component of a novel chromatin-remodeling complex, is required for cell cycle progression. Mol Cell Biol.

[CR8] Cassart C, Drogat J, Migeot V, Hermand D (2012). Distinct requirement of RNA polymerase II CTD phosphorylations in budding and fission yeast. Transcription.

[CR9] Coudreuse D, van Bakel H, Dewez M, Soutourina J, Parnell T, Vandenhaute J, Cairns B, Werner M, Hermand D (2010). A gene-specific requirement of RNA polymerase II CTD phosphorylation for sexual differentiation in *S. pombe*. Curr Biol.

[CR10] Devos M, Mommaerts E, Migeot V, van Bakel H, Hermand D (2015). Fission yeast Cdk7 controls gene expression through both its CAK and C-terminal domain kinase activities. Mol Cell Biol.

[CR11] Drogat J, Migeot V, Mommaerts E, Mullier C, Dieu M, van Bakel H, Hermand D (2012). Cdk11-cyclinL controls the assembly of the RNA polymerase II mediator complex. Cell Rep.

[CR12] Eser P, Wachutka L, Maier KC, Demel C, Boroni M, Iyer S, Cramer P, Gagneur J (2016). Determinants of RNA metabolism in the *Schizosaccharomyces pombe* genome. Mol Syst Biol.

[CR13] Estrada J, Wong F, DePace A, Gunawardena J (2016). Information integration and energy expenditure in gene regulation. Cell.

[CR14] Ganguli D, Chereji RV, Iben JR, Cole HA, Clark DJ (2014). RSC-dependent constructive and destructive interference between opposing arrays of phased nucleosomes in yeast. Genome Res.

[CR15] Garcia JF, Dumesic PA, Hartley PD, El-Samad H, Madhani HD (2010). Combinatorial, site-specific requirement for heterochromatic silencing factors in the elimination of nucleosome-free regions. Genes Dev.

[CR16] Hartley PD, Madhani HD (2009). Mechanisms that specify promoter nucleosome location and identity. Cell.

[CR17] Hsu JM, Huang J, Meluh PB, Laurent BC (2003). The yeast RSC chromatin-remodeling complex is required for kinetochore function in chromosome segregation. Mol Cell Biol.

[CR18] Huang J, Hsu JM, Laurent BC (2004). The RSC nucleosome-remodeling complex is required for Cohesin’s association with chromosome arms. Mol Cell.

[CR19] Kemmeren P, Sameith K, van de Pasch LA, Benschop JJ, Lenstra TL, Margaritis T, O’Duibhir E, Apweiler E, van Wageningen S, Ko CW (2014). Large-scale genetic perturbations reveal regulatory networks and an abundance of gene-specific repressors. Cell.

[CR20] Korber P (2012). Active nucleosome positioning beyond intrinsic biophysics is revealed by in vitro reconstitution. Biochem Soc Trans.

[CR21] Lantermann AB, Straub T, Stralfors A, Yuan GC, Ekwall K, Korber P (2009). *Schizosaccharomyces pombe* genome-wide nucleosome mapping reveals positioning mechanisms distinct from those of Saccharomyces cerevisiae. Nat Struct Mol Biol.

[CR22] Lenglez S, Hermand D, Decottignies A (2010). Genome-wide mapping of nuclear mitochondrial DNA sequences links DNA replication origins to chromosomal double-strand break formation in *Schizosaccharomyces pombe*. Genome Res.

[CR23] Li H, Hou J, Bai L, Hu C, Tong P, Kang Y, Zhao X, Shao Z (2015). Genome-wide analysis of core promoter structures in *Schizosaccharomyces pombe* with DeepCAGE. RNA Biol.

[CR24] Lieleg C, Krietenstein N, Walker M, Korber P (2015). Nucleosome positioning in yeasts: methods, maps, and mechanisms. Chromosoma.

[CR25] Materne P, Anandhakumar J, Migeot V, Soriano I, Yague-Sanz C, Hidalgo E, Mignion C, Quintales L, Antequera F, Hermand D (2015). Promoter nucleosome dynamics regulated by signalling through the CTD code. eLife.

[CR26] Materne P, Vázquez  E, Sánchez  M, Yague-Sanz C, Anandhakumar J, Migeot V, Antequera F, Hermand D (2015). Histone H2B ubiquitylation represses gametogenesis by opposing RSC-dependent chromatin remodeling at the ste11 master regulator locus. eLife.

[CR27] Monahan BJ, Villen J, Marguerat S, Bahler J, Gygi SP, Winston F (2008). Fission yeast SWI/SNF and RSC complexes show compositional and functional differences from budding yeast. Nat Struct Mol Biol.

[CR28] Parnell TJ, Huff JT, Cairns BR (2008). RSC regulates nucleosome positioning at Pol II genes and density at Pol III genes. EMBO J.

[CR29] Parnell TJ, Schlichter A, Wilson BG, Cairns BR (2015). The chromatin remodelers RSC and ISW1 display functional and chromatin-based promoter antagonism. eLife.

[CR30] Pointner J, Persson J, Prasad P, Norman-Axelsson U, Stralfors A, Khorosjutina O, Krietenstein N, Svensson JP, Ekwall K, Korber P (2012). CHD1 remodelers regulate nucleosome spacing in vitro and align nucleosomal arrays over gene coding regions in *S. pombe*. EMBO J.

[CR31] Robellet X, Fauque L, Legros P, Mollereau E, Janczarski S, Parrinello H, Desvignes JP, Thevenin M, Bernard P (2014). A genetic screen for functional partners of condensin in fission yeast. . G3 (Bethesda).

[CR32] Shim EY, Hong SJ, Oum JH, Yanez Y, Zhang Y, Lee SE (2007). RSC mobilizes nucleosomes to improve accessibility of repair machinery to the damaged chromatin. Mol Cell Biol.

[CR33] Soriano I, Quintales L, Antequera F (2013). Clustered regulatory elements at nucleosome-depleted regions punctuate a constant nucleosomal landscape in *Schizosaccharomyces pombe*. BMC Genom.

[CR34] Struhl K (1999). Fundamentally different logic of gene regulation in eukaryotes and prokaryotes. Cell.

[CR35] Struhl K, Segal E (2013). Determinants of nucleosome positioning. Nat Struct Mol Biol.

[CR36] Toselli-Mollereau E, Robellet X, Fauque L, Lemaire S, Schiklenk C, Klein C, Hocquet C, Legros P, N’Guyen L, Mouillard L (2016). Nucleosome eviction in mitosis assists condensin loading and chromosome condensation. EMBO J.

[CR37] van Werven FJ, Neuert G, Hendrick N, Lardenois A, Buratowski S, van Oudenaarden A, Primig M, Amon A (2012). Transcription of two long noncoding RNAs mediates mating-type control of gametogenesis in budding yeast. Cell.

[CR38] Wippo CJ, Israel L, Watanabe S, Hochheimer A, Peterson CL, Korber P (2011). The RSC chromatin remodelling enzyme has a unique role in directing the accurate positioning of nucleosomes. EMBO J.

[CR39] Yamada K, Hirota K, Mizuno K, Shibata T, Ohta K (2008). Essential roles of Snf21, a Swi2/Snf2 family chromatin remodeler, in fission yeast mitosis. Genes Genetic Syst.

